# Sepsis assessment in SJS/TEN: an important point overlooked?^☆^^☆☆^

**DOI:** 10.1016/j.abd.2019.06.002

**Published:** 2019-11-11

**Authors:** Ananta Khurana, Mukesh Kumar Sharma, Kabir Sardana

**Affiliations:** a Department of Dermatology, Dr. RML Hospital, PGIMER, New Delhi, India; b Department of Burns, Plastic and Maxillofacial Surgery, Dr. RML Hospital, PGIMER, New Delhi, India

Dear Editor,

We read the study “Epidermal necrolysis: SCORTEN performance in AIDS and non-AIDS patients”^1^ with interest and congratulate the authors for their important work. We would like to highlight a point regarding the utility of SCORTEN in epidermal necrolysis. The authors rightly point out that generalized infections and sepsis are the major causes of mortality in SJS/TEN patients. However, SCORTEN does not include any direct marker of the infective state. It assesses parameters that may indicate infection or just reflect the ongoing systemic inflammatory response. Unfortunately, sepsis may have already set in at the time of admission. It is essential to know the infection status at presentation, both for prognostication and for deciding specific management. A delay in diagnosis of sepsis can result in rapid progression to circulatory collapse and organ failure. Further, any form of iatrogenically induced immunosuppression may be fatal for a septic patient. Awaiting cultures may postpone important decisions and may not always give an accurate picture. Non-specific markers, such as C-reactive protein, erythrocyte sedimentation rate, total leucocyte count, and platelet count share similar concerns. An ideal marker for early sepsis diagnosis needs to be sensitive, specific, rise early in the course of sepsis, produce reliable and reproducible results, and be easy to measure in a hospital setting. The available parameter that comes closest to these criteria is probably serum procalcitonin (PCT). The reactive pattern of PCT has an onset within four hours of response to infection or injury, peaks at six hours with a plateau of eight to 24 h, then returns to baseline in two to three days.^2^ The normal levels of PCT are about 0.05 ng/mL. Higher levels, up to 0.5 ng/mL, occur in local infections, 0.5–2 ng/mL in systemic infections, 2–10 ng/mL in sepsis, and >10 ng/mL in severe sepsis.^3^

The utility of PCT for sepsis determination has been largely established in burn patients.^2^, ^4^ A similar systemic inflammatory response occurs in TEN, making differentiation from sepsis difficult. We have observed that a very high PCT value within 24 h of admission (>10 ng/mL) is a predictor of worse outcomes, irrespective of the SCORTEN value in the same time frame. Similar observations have been made by Mokline et al. in burn patients.^2^

Thus, we believe that day 0 PCT levels should be considered as an independent prognostic marker for SJS/TEN in addition to the validated parameters of SCORTEN. Further, we encourage trials specifically evaluating the role of PCT in the management of SJS/TEN.

## Financial support

None declared.

## Authors’ contribution

Ananta Khurana: Approval of the final version of the manuscript; critical literature review; effective participation in research orientation; critical manuscript review; preparation and writing of the manuscript.

Mukesh Kumar Sharma: Approval of the final version of the manuscript; critical literature review; critical manuscript review; preparation and writing of the manuscript.

Kabir Sardana: Approval of the final version of the manuscript; critical literature review; effective participation in research orientation; critical manuscript review; preparation and writing of the manuscript.

## Conflicts of interest

None declared.
